# The Challenges of Underweight and Overweight in South African Children: Are We Winning or Losing the Battle? A Systematic Review

**DOI:** 10.3390/ijerph120201156

**Published:** 2015-01-22

**Authors:** Makama Andries Monyeki, Adedapo Awotidebe, Gert L. Strydom, J. Hans de Ridder, Ramoteme Lesly Mamabolo, Han C. G. Kemper

**Affiliations:** 1 Physical Activity, Sport and Recreation Focus Area (PhASRec); North-West University; Potchefstroom 2520, South Africa; E-Mails: awotidebe.adedapo@gmail.com (A.A.); gert.strydom@nwu.ac.za (G.L.S.); hans.deridder@nwu.ac.za (J.H.R.); 2 Department of Nutrition, School of Health Sciences, University of Venda; Thohoyandou 0950, South Africa; E-Mail: ramoteme.mamabolo@univen.ac.za; 3 VU University Medical Centre, Institute for Research in Health and Care (EMGO), 1081 BT Amsterdam, The Netherlands; E-Mail: hancgkemper@upcmail.nl

**Keywords:** undernutrition, overnutrition, growth, development, functional capacity, rural, urban, South African children

## Abstract

Underweight and overweight are adverse effects of malnutrition and both are associated with negative health consequences in children and adolescents. In South Africa, the burden of economic and social disparity coexists with malnutrition in children. The purpose of this study was to review available South African studies regarding the comprehensive summary of prevalence of underweight and overweight and evaluates government policies in addressing undernutrition and overnutrition in South African children and adolescents. We searched subject-specific electronic bibliographic databases of observational studies published on malnutrition, undernutrition, overnutrition, underweight and overweight in South African boys and girls from birth to 20 years of age in studies published on or after 1990. A total of sixteen cross-sectional, three longitudinal studies and one report met the criteria for inclusion in this review. Descriptive data synthesis revealed the small number of longitudinal studies highlights the dearth of research in tracking undernutrition and overnutrition in South African children. In this review, 0.7%–66% of underweight was reported among children in rural areas compared to a 3.1%–32.4% of overweight in urban areas. All studies reported a higher rate of underweight in boys than girls who were significantly more likely to have higher body fat. The data indicated that both underweight and overweight were positively related with health-related physical activity and psychological health problems such as low activity, low fitness, low self-image and self-esteem. Numerous recommendations were made in the reviewed studies, however effective strategic programs in eradicating both underweight and overweight are minimal. It is evident from the reviewed studies that the burden of underweight and overweight are still a problem in South African children. The most highly affected by underweight are rural children, while children in urban areas in transition are faced with burden of overweight. There is little evidence to suggest that government strategic programs are effective in addressing underweight and overweight in South African children. Based on these findings, sustainable school-based feeding schemes and physical education programmes are needed for optimal benefits in children and adolescents.

## 1. Introduction

Malnutrition is both an immediate and a distant risk factor for early mortality and morbidity in children and adolescent [1,2,3]. Two burdens of malnutrition exist (undernutrition and overnutrition) and both are associated with economic inequalities and social disparities [4]. The data from South Africa 2002 National Youth Risk Behaviour survey indicated that the prevalence of underweight in children between 13–19 years was 9% and about 17% were overweight [5]. Undernutrition, which is expressed as stunting, underweight and wasting is linked to nutritional deprivation and is higher among boys in rural areas and informal settlements [4,6,7]. Similarly, overnutrition (expressed as overweight or obesity in children) is associated with sedentary lifestyles and preponderance of energy dense foods over balanced diets following increasing urbanisation and technology [8,9,10].

In line with the health promotion strategies, regional tracking of underweight and overweight distribution in children is used as a stimulus to prioritise government policies and evaluate government feeding scheme for children [11]. It has been recommended that behavioural interventions including promotion of government school feeding scheme, physical activity [12] and dietary interventions [5] should receive attention. Despite the increasing calls from the World Health Organisation for population monitoring and regional distribution of malnutrition levels in children, such studies are few in South Africa.

Studies tracking and monitoring the regional and provincial distribution of underweight and overweight in children and adolescents can assist the government in the planning of school feeding scheme policies and nutritional guidelines across the provinces. It will also facilitate the equal distribution of resources and strengthen communication action in provinces where the risks are higher.

To date no such studies are known to exist; this observation underlines the need to critically appraise all the relevant studies and integrate the findings to inform government decisions on adolescent dietary and physical activity interventions.

## 2. Objectives

The primary objective of this study was to review the available literature regarding the prevalence of undernutrition and overnutrition in South African children and adolescents. The secondary objective of the study was to evaluate government dietary intervention policies in addressing undernutrition and overnutrition in South African children and adolescents.

## 3. Methods

### 3.1. Study Inclusion Criteria

This review considered longitudinal and cross-sectional studies that determined the prevalence of underweight, stunting, wasting, overweight and obesity in children and adolescents aged 0–20 years. No provincial boundaries were set for inclusion criteria in this review. However, because of meagre resources we did not search in grey literature such as conference proceedings, unpublished studies and literature search was limited to studies published in English.

Studies were eligible for inclusion in this review if they included anthropometric measure of obesity in the form of body mass index (BMI), waist circumference (WC), sum of skinfolds-thickness, percentage body fat (%BF), waist-to-hip ratio (WHtR). Adjusted for age, sex, and ethnicity, BMI of <5th percentile is considered underweight, BMI of 5th percentile to <85th percentile is considered normal weight status, BMI of ≥85 percentile to <95th percentile is considered overweight and ≥95th percentile is considered obese [13]. Furthermore, children with waist-to-height of ≥0.5 are considered higher risk for CVD risk factor compared to those with <0.5 cm [14,15]. In addition, we also included stunting and wasting because they are also forms of malnutrition in children and adolescents and majority of included studies measured them.

### 3.2. Search Strategy

**Figure 1:**
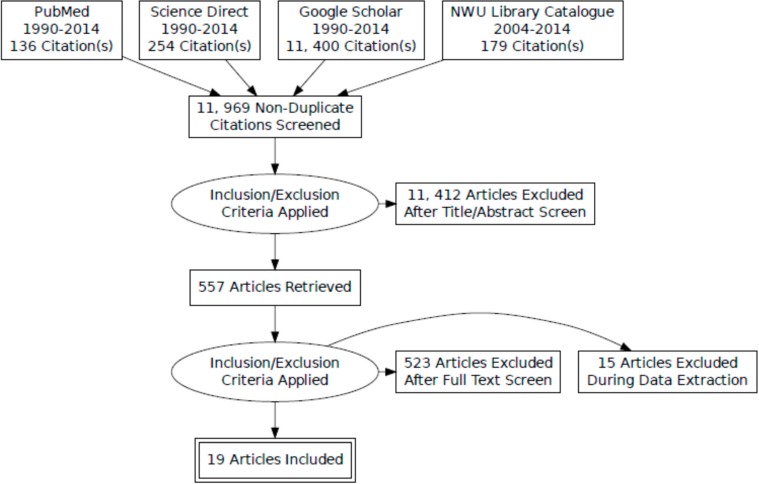
PRISMA flow chart of search strategy results.

Figure 1 show the flow chart for study selection process. To evaluate the prevalence of malnutrition in South African children and adolescents, a comprehensive electronic search was conducted for longitudinal and cross-sectional studies published between years 1990–2014 in the following subject-specific databases: PubMed, Science Direct, Google Scholar and library catalogue journals. Specific search terms relating to malnutrition, underweight, overweight, obesity, children and adolescents were developed and truncated with wildcard characters to identify eligible studies. The following terms were used: [“malnutrition” OR “obesity” OR “obes*” OR “overweight” OR underweight OR stunting OR wasting] AND [“adolescen*” OR “adolescents” OR “children*”] AND [policy implementation OR nutrition policy OR obesity policy OR food policy] AND [“South Africa”]. The date of last search was completed in 25 May 2014.

For the secondary objective of the study one report on National School Nutrition (NSNP) by the Department of Education (DoE) in 2004 [39] was found and evaluated.

### 3.3. Study Selection

Two review authors independently searched the electronic databases and screened for titles and abstracts to identify eligible studies based on the pre-specified inclusion criteria. The third review author independently confirmed the inclusion of studies and where there was a disagreement; we printed out the full copy of these studies and jointly assessed its eligibility for inclusion in the review. Where we still did not agree on the inclusion or exclusion of a study, a fourth reviewer was asked to make the final judgment. Studies were excluded if they did not meet the target age range (0–20 years), study design (observational studies), participant’s characteristics (only healthy subjects) and where data were either missing and were not reported in the selected studies.

### 3.4. Data Extraction and Analysis

Relevant data were extracted and presented as description of characteristics of included studies (Table 1). Data were extracted independently to collect information on study sources, settings, participant’s characteristics, study design, anthropometric measurements, and body weight categories. Where data was not clear or contradictory, consensus was reached through discussion. Data were analysed descriptively and summaries presented as means and standard deviations for BMI and proportions for underweight, stunting, wasting, overweight and obesity.

**Table 1:** The 19 selected studies with prevalence of malnutrition as measured by underweight, stunting, wasting, overweight and obesity.

	Participants	Study Design	Growth Reference Standards	Outcome and Main Findings
Shisana *et al.* [16]	South African National Health and Nutrition Examination Survey (0–14 years (yrs): N = 8629) (SANHANES-I).	National survey	WHO Child Growth Standard for 0–5 years. The WHO Reference 2007 for 5–19 years.	Outcome: proportions of stunting, wasting, underweight, overweight and obesity
				**Stunting Boys**: 0–3 yrs = 26.9%; 4–6 yrs = 13.5%; 7–9 yrs = 10.0%; 10–14 yrs = 15.2%
				**Stunting Girls**:0–3 yrs = 25.9%; 4–6 yrs = 9.5%; 7–9 yrs = 8.7%; 10–14 yrs = 10.1%
				**Wasting Boys**: 0–3 yrs = 3.8%; 4–6 yrs = 2.6%; 7–9 yrs = 2.4%; 10–14 yrs = 5.6%
				**Wasting Girls**: 0–3 yrs = 1.5%; 4–6 yrs = 1.0%; 7–9 yrs = 1.2%; 10–14 yrs = 2.5%
				**Underweight Boys**: 0–3 yrs = 8.2%; 4–6 yrs = 5.4%; 7–9 yrs = 8.6%; 10–14 yrs = 0%
				**Underweight Girls**: 0–3 yrs = 3.6%; 4–6 yrs = 3.2%; 7–9 yrs = 4.0%; 10–14 yrs = 3.2%
				**Overweight: Boys**:17.5% (2–5 yrs); 4.5% (6–9 yrs);7.5%(10–14 yrs)
				**Girls**: 18.9% (2–5 yrs); 12.3% (6–9 yrs); 16.7% (10–14 yrs)
				**Obesity: Boys**: 4.4% (2–5 yrs); 2.7% (6–9 yrs); 2.7% 10–14 yrs)
				**Girls**: 4.9% (2–5 yrs); 4.1% (6–9 yrs); 5.6% (10–14 yrs).
Mamabolo *et al.* [17]	181 (70 boys & 111 girls) aged 13–20 years from township in Potchefstroom, **North West province**.	Cross-sectional study design.	WHO Ref of 2007 for prevalence of stunting and underweight. BMI age-adjusted cut off points described Cole 2000 to estimate overweight and obesity.	Outcome: proportions of stunting, underweight, overweight and obesity
				**Stunting**: 17.1% Boys; 18.9% Girls
				**Underweight**: 11.4% Boys; 0% Girls
				**Overweight & Obesity**: 4.1% Boys; 9.9% Girls.
Monyeki *et al.* [12]	256 (100 Boys; 156 Girls) aged 14 years from 4 township schools and 2 urban schools in Potchefstroom, **North West province**.	Cross-sectional	Age and sex-specific cut points described by Cole 2000 & 2007 to estimate, underweight, overweight and obesity.	Outcome: proportion of underweight, overweight and obesity
				**Underweight**: 44% Boys; 30.7% Girls
				**Overweight & Obesity**: 8% Boys; 17.3% Girls
Toriola & Monyeki [18]	283 (111 Boys; 172 Girls) aged 14 years from 4 township schools in Potchefstroom **North West province**.	Cross-sectional	Age-specific BMI to estimate, underweight, overweight and obesity described by Cole 2000 & 2007.	Outcome: proportion of underweight, overweight and obesity
				**Underweight**: 34.2% Boys; 26.6% Girls
				**Overweight**: 17.1% Boys; 32.4% Girls
Tathian *et al.* [19]	959 female learners in 31 primary schools from **KwaZulu Natal**.	Cross-sectional	WHO/NCHS for proportion of stunting and underweight. Age and gender-specific cut off point for overweight & obesity described by Cole 2000.	Outcome: proportion of stunting, underweight, overweight & obesity
				**Stunting**: 9.2%
				**Underweight**: 4%
				**Overweight**: 9%
				**Obesity**: 3.8%
Toriola *et al.* [20]	1172 (541 Boys & 631 Girls) Black school children aged 10–16 years from two settlements in **Limpopo province**.	Cross-sectional	CDC BMI charts to classify participant’s under-weight, overweight and obesity status.	Outcome: proportion of underweight, overweight and obesity.
				**Underweight**: 4.6% Boys; 5.2% Girls
				**Overweight**: 9.1% Boys; 11.0% Girls
				**Obesity**: 5.5% Boys; 4.4% Girls
Puckree *et al.* [21]	120 predominantly Indian children aged 10–12 years from six public schools in **urban district of KwaZulu Natal**.	Cross-sectional	WHO guidelines and advice from local paediatrician to estimate underweight and overweight.	Outcome: proportion of underweight and overweight
				**Underweight**: 66% (Black 15%; Indian 51%
				**Overweight**: 5.03%
Mamabolo *et al.* [22]	162 children from rural villages in **Limpopo province**.	Prospective cohort study	WHO/NCHS for stunting, wasting and under-weight. IOTF reference for overweight and obesity.	Outcome: proportion of stunting, wasting, underweight, overweight and obesity
				**Stunting**: 48%
				**Underweight**: 10%
				**Wasting**: 1%
				**Overweight**: 22%
				**Obese**: 24%
Kimani-Murage *et al.* [4]	3511 children and adolescents aged 1–20 years from **Mpumalanga province**.	Cross-sectional	2006 WHO standard for 0–4 years and 1977 NCHS/WHO for 5–17 years to estimate stunting, wasting and underweight. IOTF BMI age and sex specific for overweight and obesity in 2–17 years.	Outcome: proportion of stunting, wasting, underweight, overweight & obesity
				**Underweight**: 18% (1–4 yrs); 5% (5–9 yrs); 7% (10–14 yrs); 6% (15–20 yrs)
				**Stunting**: 10% (1–4 yrs); 6% (5–9 yrs); 7% (10–14 yrs); 8% (15–20 yrs)
				**Wasting**: 7% (1–4 yrs); 6% (5–9 yrs); 0% (10–14 yrs); 0% (15–20 yrs)
				**Overweight**: 7% (1–4 yrs); 4% (5–9 yrs); 6% (10–14 yrs); 8% (15–20 yrs)
				**Obesity**: 1% (1–4 yrs); 1% (5–9 yrs); 2% (10–14 yrs); 4% (15–20 yrs)
Craig *et al.* [23]	1519 children in grade 1, 5 and 7 with a mean ages 7, 11 and 15 years in **KwaZulu Natal province**.	Cross-sectional	BMI-for-age using WHO 2007, Cole-IOTF, & 1977 NCHS/WHO to estimate underweight, overweight and obesity.	Outcome: proportion of stunting, wasting, underweight, overweight & obesity
				**Underweight:**
				(a) WHO 2007: 3.4% Boy; 1.2 Girl, 7 yrs; 5.2% Boy; 1.9% Girl, 11 yrs; 6.2% Boy; 1.9% Girls, 15 yrs
				(b) Cole-IOTF: 16% Boy; 15.1% Girl, 7 yrs; 12.9% Boy; 12.3% Girl, 11 yrs; 15.8% Boys; 8.2% Girls, 15 yrs
				(c) NCHS/WHO: 6.2% Boy; 2.8% Girl; 7 yrs; 3.9% Boy; 1.9% Girl, 11 yrs; 7.6% Boy; 1.0% Girls, 15 yrs
				**Overweight:**
				(a) WHO 2007: 8.4% Boy; 11.6% Girl, 7 yrs; 4.7% Boy; 11.9% Girl, 11 yrs; 5.7% Boy; 17.8% Girls, 15 yrs
				(b) Cole-IOTF: 3.0% Boy; 7.2% Girl, 7 yrs; 3.0% Boy; 8.6% Girl, 11 yrs; 4.9% Boy; 17.4% Girls, 15 yrs
				(c) NCHS/WHO: 9.6% Boy; 14.6% Girl, 7 yrs; 3.5% Boy; 6.0% Girl, 11 yrs; 4.4% Boy; 9.9% Girls, 15 yrs
				**Obesity:**
				(a) WHO 2007: 0.8% Boy, 2.0% Girl, 7 yrs; 3.4% Boy; 1.5% Girl, 11 yrs; 2.3% Boy; 8.0% Girls, 15 yrs
				(b) Cole-IOTF: 0.4%; 2.0% Girl, 7 yrs; 2.2% Boy; 1.1% Girl, 11 yrs; 1.2% Boy; 5.3% Girl, 11 yrs
				(c) NCHS/WHO: 3.1% Boy; 7.7% Girl, 7 yrs; 0.9% Boy; 1.1% Girl, 11 yrs; 0.6% Boy; 2.3% Girl 15 yrs
26.Jacobs & De Ridder [24]	168 (79 Boys & 89 Girls) Black South African children in rural areas from the **North West province**.	Cross-sectional	ACSM 2006 to estimate underweight, over-weight and obesity.	Outcome: proportion of underweight, overweight and obesity
				**Underweight**: 19% Boys; 11% Girls
				**Overweight and obesity**: 0% Boys; 7% Girls
Ginsburg *et al.* [25]	1613 (773 Boys & 840 Girls) of cohort South African urban children in Gauteng province.	Longitudinal	BMI Z-score using WHO reference to estimate mean BMI z-score. Age and sex-specific BMI by Cole 2000 & 2007 for underweight, over-weight and obesity.	Outcome: Mean BMI z-score and proportion of underweight, overweight & obesity
				**Mean BMI z**-score: −0.47 Boys and 0.32 Girls
				**Underweight**: 20.3% Boys; 9.6 Girls
				**Overweight**: 5.4% Boys; 17.5 Girls
				**Obese**: 2.5% Boys; 7.5% Girls
Reddy *et al.* [5]	9224 school children aged 13–19 from grade 8–11 selected from public schools in **all nine provinces**.	National survey	Prevalence of underweight was based on WHO/NCHS. Overweight and obesity was based on Cole-IOTF.	Outcome: proportion of underweight, overweight and obesity
				**Underweight**: 9% National (15.6% Boys & 3.9% Girls)
				**Overweight**: 16.9% National (6.9% Boys; 24.5% Girls)
				**Obese**:4% National (2.2% Boys; 5.3% Girls)
Bosman *et al.* [26]	1512 (52.8% Boys &47.2% Girls) children aged 1–5 years using data from 1999 NFCS database.	National survey	1977 NCHS, 2000 CDC and 2006 WHO growth standard were used to estimate stunting, wasting underweight, overweight and obesity.	Outcome: proportion of stunting, wasting, underweight, overweight and obesity
				**Stunting**: 2006 WHO Standard: 20.1%; 1977 NCHS: 17.1%; 2000 CDC: 14.2%
				**Wasting**: 2006 WHO standard: 10%; 1977 NCHS: 15%; 2000 CDC: 15%
				**Underweight**: 2006 WHO standard: 6.8%; 1977 NCHS: 9.7%; 2000 CDC: 9.9%
				**Overweight**: 2006 WHO standard: 20.6%; 1977 NCHS: 13.0%; 2000 CDC: 16.7%
				**Obese**: 2006 WHO standard: 9.5%; 1977 NCHS: 5.9%; 2000 CDC: 6.3%
Jinabhai *et al.* [9]	2398 Boys and 2924 Girls Black teenagers aged 13–18 years in the first South African Youth Risk Behaviour Survey (2002).	National survey	The NCHS and CDC used data from NHANES II which WHO recommended for international use to estimate stunting & underweight. Age-dependent BMI cut off by Cole 2000 for overweight and obesity.	Outcome: proportion of stunting, underweight and overweight
				**Stunting**: 21.9% Boys; 9.4% Girls
				**Underweight**: 18.4% Boys; 2.6% Girls
				**Overweight**:4.2% Boys; 20.9% Girls
Jinabhai *et al.* [27]	802 children in grade 3 aged 8 and 11 years from 11 schools in rural district of **Kwazulu Natal**.	Cross-sectional	NCHS was used to estimate prevalence of stunting. Calculations for overweight and obesity were based on WHO (1995) and Cole-IOTF.	Outcome: prevalence of stunting, overweight and obesity
				**Stunting**:31%–100% mild; 3%–25% moderate stunting; 0.6% severe stunting
				**Overweight**: 4.1% IOTF cut off point; 6.0% WHO definition
				**Obesity**: 0.6% IOTF; 0.9% WHO definition
Monyeki *et al.* [28]	1335 (684 Boys & 651 Girls) rural children aged 3–10 years from Ellisras, rural **Limpopo province**.	Cross-sectional	NHANES I & II or NCHS reference cut off point to determine prevalence of stunting and wasting.	Outcome: prevalence of stunting, wasting and WAZ (underweight)
				**WAZ (underweight)**: 20.9%–64.3% (highest in 9–11 yrs)
				**HAZ (stunting)**: 3.7%–28.6% (highest in 10–11 yrs)
				**WHZ (wasting)**: 27.7%–54.6% (highest in 3–3.9 yrs)
Monyeki *et al.* [29]	1339 (687 Boys & 652 Girls) children aged 3–10 years from Ellisras, rural **Limpopo province**.	Cross-sectional	WAZ, HAZ and WHZ were determined using NHANES III.	Outcome: prevalence of stunting and wasting
				**Stunting**: 19.9%–51.0%
				**Wasting**: 22.8%–39.9%
Labadarios *et al.* [7]	A national representative of 2613 children aged 1–9 years.	National survey	NCHS reference median to determine WAZ, HAZ and WHZ. Standard BMI cut off point for overweight and obesity.	Outcome: prevalence of stunting, underweight, wasting, overweight and obesity
				**Stunting**: 21.6% National; 30.6% Commercial Farm; 16% Formal Urban; 19.3% Informal Urban; 16.7% Urban; 26.5% Rural
				**Underweight**: 10.3% National; 18.1% Commercial Farm; 7.8% Formal Urban; 7.6% Informal Urban; 7.7% Urban; 12.8% Rural
				**Wasting**: 3.7% National; 4.2% Commercial Farm; 2.6% Formal Urban; 2.1% Informal Urban; 2.4% Urban; 4.9% Rural
				**Overweight**: 12.1% National; 7.2% Farm; 13.9% Formal Urban; 7.5% Informal Urban; 11.6% Rural; 12.5% Urban
				**Obese**: 5.0% National; 3.5% Commercial Farm; 6.2% Formal Urban; 5.9% Informal Urban; 3.7% Rural; 6.1% Urban

## 4. Results

Table 1 illustrates the selected 19 studies with prevalence of malnutrition as measured by underweight, stunting, wasting, overweight and obesity [4,5,7,9,12,16,17,18,19,20,21,22,23,24,25,26,27,28,29]. Sixteen cross-sectional studies and three longitudinal studies met the inclusion criteria and were included in the review. Six studies [4,5,9,17,20,23] were included because the lower limits of the age range of the participants overlapped with the target age range in our review.

## 5. National Demographics of Included Studies

Four studies each were conducted in North West province [12,17,18,24]; KwaZulu-Natal province [19,21,23,27] and Limpopo province [20,22,28,29]. One study each from Gauteng [25] and Mpumalanga [4] provinces. The remaining five studies were national health surveys [5,7,9,16,26].

## 6. Measurements

### 6.1. Assessment of Underweight, Stunting, and Wasting

For estimating underweight, stunting and wasting in children, five studies [4,5,7,19,22] used National Centre for Health Statistics (NCHS)/ World Health Organisation BMI Z-scores (‒2 SD) to estimate underweight (BMI for age; BAZ), stunting (Height for age; HAZ) and wasting (Weight for height; WHZ) in children and adolescents. Three studies [12,18,25] used method described in Cole *et al.* (2007, 2000) to estimate underweight in targeted participants. The United States National Health and Nutritional Examination Survey (NHANES) I, II, & III criteria were used in two studies [28,29] to estimate under-nutrition in children. Two studies used CDC BMI growth chart [9,20] and in one study [27], criteria discussed in WHO (1995) was used to estimate malnutrition. Only two studies used WHO Reference 2007 to estimate underweight and stunting [16,17] and one study used 2006 WHO standard in children less than 5 years and 1977 NCHS/WHO for older children less than 18 years [4]. Finally, two studies compared the WHO 2007, IOTF, NCHS/WHO, CDC 2000 and 2006 WHO standard to estimate malnutrition in children [23,26].

### 6.2. Assessment of Overweight and Obesity

In ninety percent of the studies reviewed, the body mass index (BMI) was used to estimate the proportion of youth in underweight, overweight and obese weight status categories. Eight studies [9,12,16,17,18,19,25,27] used the BMI cut-off point criteria described by Cole *et al.* (2007, 2001), two studies (9, 20) used Centre for Disease Control and Prevention (CDC) BMI growth chart, and three [5,22,23] studies estimated the BMI from the International Obesity Task Force (IOTF). Three studies [4,23,26] compared the WHO (2007) reference, IOTF, WHO/NCHS, CDC 2000 and 2006 WHO standard criteria in estimating the weight status in South African children and adolescents.

### 6.3. Prevalence of Underweight

The results of studies using [4,5,7,9,22] NCHS/WHO references showed that the prevalence of underweight in children was between 4%–19%. All studies showed that higher proportion of boys are underweight compared to girls. In studies that described the national demographics, prevalence of underweight was highest among Coloured children (Coloured = 10.6%; Black = 9.5%; White = 1.9%) and higher in rural (12.8%) compared to urban dwellers (7.7%). Similar results were observed in studies that used CDC references with a prevalence of underweight ranging from 4.6%–19%. However, higher proportions of children were estimated to be underweight using Cole-2007 and NHANES references. For example, the prevalence of underweight in Monyeki *et al.* [28] ranged from 20.9%–64.3%. In studies that compared the different references of estimating underweight, Cole-IOTF estimated higher prevalence of underweight in children compared to WHO 2007, NCHS/WHO and CDC references.

### 6.4. Prevalence of Stunting

In those studies [4,5,7,9,22] that estimated the prevalence of stunting using the NCHS reference, the prevalence ranged from 6% to 30.6%. Children staying in commercial farm are more stunted (30.6%) compared to children in rural (26.5%) and formal urban setting (16%). In contrast, another study [7] found girls to be more stunted (18.9%) compared to boys (17.1%). In a study [26] that compared different methods of estimating stunting, 2006 WHO standard reference estimated higher proportion of children with stunting (20.1%) than 1977 NCHS (17.1%) and CDC 2000 (14.2%).

### 6.5. Prevalence of Wasting

Only five studies [16,22,26,28,29] estimated the prevalence of wasting in children. Studies [28,29] using the NHANES I, II, & III criteria estimated the higher proportion of children with wasting with a prevalence of 22.8% to 54.6%. Young children between the ages of 1–4 years are more stunted than children between the age of 5–9 years and no wasting found in those ≥10 years [8]). The prevalence of wasting is higher in rural (4.9%) and commercial farm children (4.2%) compared to children in informal urban (2.1%) and urban (2.6%) dwellers. In the study [26] that compared different methods of estimating wasting, a similar proportion (15.0%) of children were estimated for wasting using 1977 NCHS and 2000 CDC reference compared to 10.0% of children using 2006 WHO standard.

### 6.6. Prevalence of Overweight

Four studies [4,5,7,9] used NCHS/WHO reference to estimate the prevalence of overweight in children and adolescents. The prevalence of overweight was 4% to 24.5%. Using Cole-2007 the prevalence ranged from 5.4% to 32.4% and between 0% to 11% using CDC growth charts. Craig *et al.* [23] compared the different methods of estimating overweight. The WHO 2007 reference and NCHS/WHO estimation were similar in proportion of children with overweight compared to Cole-IOTF. In contrast, 1977 NCHS estimated the least proportion of children with overweight compared to 2006 WHO standard and 2000 CDC [26]. In all studies, higher proportions of girls are more overweight compared to boys. The national distribution shows that white children are more overweight (23.4%) compared to black children (16.6%) and coloured children (13%). In addition, national prevalence of overweight was 12.1% and the proportion is higher among children from formal urban and urban setting (13.9%, 12.5%) compared to children from commercial farm (7.2%) and rural area (11.6%).

### 6.7. Prevalence of Obesity

The prevalence of obesity among children in studies that used NCHS/WHO reference ranged from 1% to 6.4%. Two studies [12,25] used Cole-2007 reference and data indicated a prevalence of 2.5% to 17.3% in children. However, the prevalence was much higher in a study that used IOTF reference with a prevalence of 24% [22]. Regarding different methods of estimating obesity, proportions of obesity was higher using 2006 WHO standard (9.5%) compared to 2000 CDC (6.3%) and 1977 NCHS (5.9%). The results of all the studies show that higher proportions of girls are more obese compared to boys. In one that surveyed the national prevalence of malnutrition in South African children, the finding showed that white children had the higher prevalence of obesity (6.4%) compared to black (3.8%) and coloured children (3.3%). National prevalence of obesity was 5.0% and higher proportion from urban (6.1%) compared to rural dwellers (3.7%). The result showed that using NCHS/WHO underestimated the prevalence of obesity in children and adolescents.

### 6.8. The Trend in the Prevalence of Malnutrition in South African Children

According to studies that used NCHS/WHO, CDC and WHO criteria, the prevalence of underweight in children and adolescent showed a decline from approximately 10% between 1999 and 2002 to approximately 5.5% in 2010. Regarding stunting, for studies [23,26] using NCHS/WHO and 2006 WHO standard, the prevalence of stunting was observed to have declined from above 20% in 1999 to 9.2% in another that used the same criteria in 2011 [5]. From this review, the trend in the prevalence of wasting was mixed and cannot be ascertained. All data collected in late 1990s using different criteria showed that prevalence was as low as 3.7% using NCHS reference and above 40% in one study that used NHANES reference. However, a recent prevalence of wasting in one study was between 6% and 7% [4]). To this end, because of the overlap in the prevalence between 1990s and 2007, it is difficult to describe the trend in prevalence of wasting. The prevalence of overweight in children was observed to have remained the same from 1999 to 2002. For example, the national prevalence of overweight in South African children was estimated to 12% to 20.6% in 1999 and 16.9% to 20.8% in 2002. The recent finding from cross-sectional data in 2011 showed an overall prevalence of overweight and obesity was estimated to be 17.3% [12]. Similar pattern was also observed for the prevalence of obesity in South African children. The prevalence of obesity using different criteria was estimated at 5% to 9.5% in 1999 and 4% and 5% in 2002 and 2010 respectively. Based on these criteria, though the prevalence of overweight and obesity in children is still high, it has remained steady in the last 10 years.

## 7. Discussion

This review highlights the burden and trend of undernutrition (underweight, stunting and wasting) and overnutrition (overweight and obesity) in South African children aged 0–20 years. The findings showed that high proportions (4%–19%) of children are underweight, higher in boys and more predominantly in rural areas. Similarly, the prevalence of stunting and wasting is higher in younger boys and among rural dwellers. In contrast, the prevalence of overweight and obesity is higher among girls and urban children compared to children in rural areas. Regarding the trend of undernutrition and over nutrition in studies reviewed, there was a decline in the prevalence of underweight and stunting, but the prevalence of overweight and obesity in children has stabilised, though still high.

In South Africa, notable government strategy is in place to address the prevalence of malnutrition in children [7,30]. Thus, the observed decline in the prevalence of underweight and stunting might be due to National Nutrition-Specific Intervention (NSNP) with coverage of around 5 million children mainly from low socio-economic background. Also, the finding from a systematic analysis of population representative data from 144 countries showed an improvement in nutritional status of children in sub-Saharan Africa [11]. However, higher proportion of underweight and stunting in children living in rural areas is consistent with associated food insecurity and household poverty in poorer communities [31]. It could also be due to delayed pubertal development observed in children living in rural areas compared to their counterparts in the city [32].

Regarding overweight and obesity, the prevalence is high among the South African children, but data showed that the trend has been stabilised in the last couple of years. The high prevalence of overweight and obesity in children is a public health dilemma because they are linked to metabolic syndromes in adult life including cancer, stroke, coronary heart disease, type 2 diabetes, among others [33]. All the studies reviewed observed that prevalence of overweight and obesity was higher in girls compared to boys and overall prevalence was higher among urban children. This finding may reflect preponderance of fast food outlets and increasing access to consumption of energy-dense foods in and around urban schools [8,9]. In addition, children in urban areas are increasingly engaging in sedentary activities including TV viewing which, has been shown to be accompanied with junk foods consumption [34], which is associated with escalating burden of excess body fat [35]. Very important is the use of evidence-based physical activity intervention to combat childhood obesity, especially in a country like South Africa which is a country with extreme diversity, not only in population and ethnic groupings, but also with regard to socio-economic status [41].

Tracking the prevalence of malnutrition has important public health perspective for promoting nutritional status in children. This review showed that different sex-and-age specific BMI references and charts (WHO 2007 reference, 2006 WHO Growth standards, Cole-IOTF 2000/2007, CDC 2000, WHO, 1995, 1977 WHO/NCHS and NHANES I, II, III) were used to estimate nutritional status in children. Thus, it is difficult to interpret findings across studies for tracking the trend and burden of malnutrition in children because of variations in reference standards used across studies. The data across the studies showed that Cole-IOTF and 2006 WHO Growth standard overestimated the prevalence of underweight, stunting and overweight compared to other reference charts. This finding is consistent with a finding from review studies among Iranian children where IOTF and WHO definitions overestimated the prevalence of overweight [36]. However, in our study we found that IOTF overestimated obesity and this finding is different from Kelishadi *et al.* [36] that found IOTF and WHO references to overestimate the prevalence of obesity among Iranian children. This difference can be explained by the fact that some of the BMI references and charts lack representativeness and are not applicable in some settings from developing countries [37]. To track unhealthy nutritional status in children, Wang [37] opined that a single sex-and-age BMI reference cut-off point must be validated to increase the generalizability of findings specific to that country. In addition to the limitations of reference growth standards to estimate the burden of malnutrition in children, another important limitation is related to sample size and representativeness of study sample in the reviewed studies. It was clear from the reviewed national studies [4,5,16,23], that the prevalence of underweight and overweight was lower compared to some regional studies with small sample sizes in which an overestimation of prevalence of underweight and overweight were reported.

In this study, we did not review various government strategies to address unhealthy nutritional status among South African children. Though, a specific-nutrition strategy is in place, it is characterised with several challenges.

## 8. Government Interventions in Addressing Malnutrition in Children

An estimated 7.4 million children aged 3 years or less die each year of malnutrition with highest proportion in sub-Saharan Africa [38]. For this reason, there are increasing calls to scale up interventions and strategies in addressing malnutrition aimed at improving health of children and adolescents. South Africa is experiencing economic growth, it is also a country characterised by extreme wealth and poverty. In response, shortly after independence in 1994, South African government initiated a Primary School Nutrition Programme (PSNP) under the purview of Department of Health (DoH) to promote and advocate for malnutrition control, nutritional education and dietary guidelines in children from low socioeconomic status [7]. Since inception in 1994 to 2002, through the government school feeding scheme, the programme has benefited approximately 5 million children and with a government expenditure of about R4 billion. The programme was renamed National School Nutrition (NSNP) and implementation transferred to the Department of Education (DoE) in 2004 to strengthen community action and buy in. The evaluation of the programme in 2001 found that provincial governments wanted to do many things at a time for political gains thereby losing sight of the quality of the programme [39]. Thus, the programme then was characterised by unequal distribution of resources especially to schools not within the radar of programme’s objectives, poor adherence to dietary guidelines and lack of proper hygiene [39]. Similarly, findings from the evaluation of NSNP programmes in Limpopo and Eastern Cape provinces showed that most beneficiaries are children from low socio-economic status, but there is a wide gap on what to be done by those implementing the programme in terms of nutritional guidelines and facilities.

As a point of departure, global strategy on diet recommended the recognition of the burden of unhealthy diets and physical inactivity and efforts must be put in place to in increase the opportunities for developing, strengthening and implementing culturally-appropriate policies and actions to improve diets and physical activity in children [40]. The combat against malnutrition in a third world country like South Africa have many challenges and especially the health care system is challenged with numerous complexities and financial constraints [41]. In brief, these should be promotion of national dietary guidelines, multi-sectorial and multidisciplinary collaborations, provision of adequate resources, implementation by relevant ministries and periodical monitoring and evaluation of diet and physical activity programmes.

## 9. Public Health and Future Implications Messages

This review carries important implications for policy makers in the field of public health regarding the magnitude of both undernutrition and overnutrition in children and adolescents. As such, this review may provide policy makers with valuable scientific information for strategic intervention program to combat both undernutrition and overnutrition so that the government can achieve the set health goals by the 2013 South African National Development Plan (SANDP, 2013) [42]. Given the variation in reference standards, there is a need to develop a standardised reference standard which will work best for specific settings taking into consideration the social, ethnic, economic and dietary variations in children.

## 10. Conclusions

In summary, it is evident from the reviewed studies that the burden of undernutrition and overnutrition are high among South African children. Children from the rural areas are mostly affected by underweight and stunting while children in urban areas because of nutritional transition are faced with burden of overweight and obesity. There is little evidence to suggest that government strategic programs are effective in addressing underweight and overweight in South African children. Based on these findings, there is a need for introduction of an effective 60 minutes per day (four hour per week) intensive physical activity in the school syllabi [43] and government need to revisit its school-feeding scheme for optimal benefits. There is a need to scale up robust longitudinal studies to address gaps in government programmes in all provinces of South Africa with a view to promoting adolescent health in South Africa.

## References

[1] World Health Organisation (WHO) Children: Reducing Mortality.

[2] Horton R. (2008). Maternal and child undernutrition: An urgent opportunity. Lancet.

[3] Blake R., Allen L., Bhutta Z., Caulfield L., de Onis M., Ezzati M., Mathers C., Rivera J. (2008). Maternal and child undernutrition: Global and regional exposures and health consequences. Lancet.

[4] Kimani-Murage E., Kahn K., Pettifor J., Tollman S., Dunger D., Gomez-Olive X., Norris S. (2010). The prevalence of stunting, overweight and obesity, and metabolic disease risk in rural South African children. BMC Public Health.

[5] Reddy S., Resnicow K., James S., Kambaran N., Omardien R., Mbewu A. (2008). Underweight, overweight and obesity among South African adolescents: Results of the 2002 National Youth Risk Bevaviour Survey. Public Health Nutr..

[6] Monyeki K.D., Kemper H.C., Makgae P.J. (2008). Relationship between fat patterns, physical fitness and blood pressure of rural South African children: Ellisras longitudinal growth and health study. J. Hum. Hypertens..

[7] Labadarios D., Steyn N., Maunder E., MacIntryre U., Gericke G., Swart R., Huskisson J., Dannhauser A., Vorster H.H., Nesmvuni A.E., Nel J.H. (2005). The national food consumption survey (NFCS): South Africa, 1999. Public Health Nutr..

[8] World Health Organisation (WHO) Obesity and Overweight.

[9] Jinabhai C., Taylor M., Reddy P., Monyeki D., Kamabaran N., Omardien R., Sullivan K. (2007). Sex differences in under and over nutrition among school-going Black teenagers in South Africa: An uneven nutrition trajectory. Trop. Med. Int. Health.

[10] Vorster H., Venter C., Wissing M., Margetts B. (2005). The nutrition and health transition in the North West province of South Africa: A review of the THUSA (Transition and Health during Urbanisation of South Africans) study. Public Health Nutr..

[11] Stevens G., Finucane M., Paciorek C., Flaxman S., White R., Donner A., Ezzati M. (2012). Nutrition Impact Model Study Group. Trends in mild, moderate, and severe stunting and underweight, and progress towards MDG 1 in 141 developing countries: A systematic analysis of population representative data. Lancet.

[12] Monyeki M.A., Neetens R., Moss S.J., Twisk J. (2012). The relationships between body composition and physical fitness in 14 year old adolescents residing withing the Tlokwe local municipality, SA: The PAHL study. BMC.

[13] Lawlor A., Benfield L., Logue J., Tilling K., Howe D., Fraser A., Cherry L., Watt P., Ness A.R., Smith G.D. (2010). Association between general and central adiposity in childhood, and change in these with cardiovascular risk factors in adolescence: Prospective cohort study. Brit. Med. J..

[14] Garnett S.P., Baur L.A., Cowell C.T. (2008). Waist-to-height ratio: A simple option for determining excess central adiposity in young people. Int. J. Obes..

[15] Savva S.C., Tornaritis M., Savva M.E., Kourides Y., Panagi A., Georgiou C., Kafatos A. (2000). Waist circumference and waist-to-height ratio are better predictors of cardiovascular risk factors in children than body mass index. Int. J. Obes..

[16] Shisana O., Labadarios D., Rehle T., Simbayi I., Zuma K., Dhansay A., Reddy P., Parker W., Hoosain E., Naidoo P. (2014). South African National Health and Nutrition Examination Survey (SANHANES-I).

[17] Mamabolo R., Berti C., Monyeki M., Kruger S. (2014). Association between insulin-like growth factor-1, measures of overnutrition and undernutrition and insulin resistance in Black adolescents living in the North-West Province, South Africa. Amer. J. Biol..

[18] Toriola O., Monyeki M. (2012). Health-related fitness, body composition and physical activity status among adolescent learners: The PAHL study. AJPHERD.

[19] Tathian N., Moodley I., Mubaiwa V., Denny L., Taylor M. (2013). South Africa’s nutritional transition: Overweight, obesity, underweight and stunting in female primary school learners in rural KwaZulu-Natal, South Africa. South Afr. Med. J..

[20] Toriola A.L., Moselakgomo V., Shaw B., Goon D. (2012). Overweight, obesity and underweight in rural black South African children. South Afr. J. Clin. Nutr..

[21] Puckree T., Naidoo P., Pillay P., Naidoo T. (2011). Underweight and overweight in primary school children in eThekwini district in Kwazulu-natal, South Africa. Afr. J. Prim. Health Care Fam. Med..

[22] Mamabolo R., Alberts M., Steyn N., Waal H., Levitt N. (2005). Prevalence and determinants of stunting and overweight in 3-year-old black South African children residing in the central region of Limpopo provine. South Afr. Public Health Nutr..

[23] Craig E., Reilly J., Bland R. (2012). Body fatness or anthropometry for assessment of unhealthy weight status: Comparison between methods in South African children and adolescents. Public Health Nutr..

[24] Jacobs S., de Ridder J. (2012). Prevalence of overweight and underweight among black South African children from rural areas in the North-West province. South Afr. J. Res. Sport Phys. Educ. Recreat..

[25] Gimsburg C., Griffiths P., Richter L., Norris S. (2013). Residential mobility, socioeconmic context and body mass index in a cohort urban South African adolescents. Health Place.

[26] Bosman L., Herselman M., Kruger S., Labadarios D. (2011). Secondary analysis of anthropometric data from a South African national food consumption survey, using growth reference standard. Maternal Child Health J..

[27] Jinabhai C., Taylor M., Sullivan K. (2003). Implications of the prevalence of stunting, overweight and obesity amongst South African promary school children: A possible nutritional transition?. Eur. J. Clin. Nutr..

[28] Monyeki K., Cameron N., Getz B. (2000). Growth and nutritional status of rural South African children 3–10 years old: The Ellisras growth study. Amer. J. Hum. Biol..

[29] Monyeki K., de Ridder J., Toriola A., Steyn N., van Lenthe F., Griebenauw L. Physical Growth and Socio-Economic Status of South African rural children Aged 3–10 Years. Proceedings of the 6th International Conference in Kinanthropometry.

[30] Kallman K. (2005). Food for Thought: A Review of the National School Nutrition Programme.

[31] Labadarios D., Mchiza Z., Steyn N., Gericke G., Maunder E., Davids Y., Parker W. (2011). Food security in South Africa: A review of national surveys. Bull. WHO.

[32] Marrodan M.D., Mesa M.S., Arechiga J., Perez-Magdaleno A. (2000). Trend in menarcheal age in Spain: Rural and urban comparison during a recent period. Ann. Hum. Biol..

[33] Allender S., Rayner M. (2007). The burden of overweight and obesity-related ill health in the UK. Obes. Rev..

[34] Taveras E., Sandora T., Shih M., Ross-Degnan D., Goldman D., Gillman M. (2006). The association of television and video viewing with fast food intake by preschool-age children. Obesity (Silver Spring).

[35] Latt E., Maestu J., Raask T., Rubin D., Purge P., Saar M., Utsal L., Jürimäe J., Maasalu K., Jürimäe T. (2013). Association of physical activity to cardiovascular fitness and fatness in 12–13 year-old boys in different weight status. J. Public Health.

[36] Kelishadi R., Haghdoost A., Sadeghirad B., Khajehkazemi R. (2014). Trend in the prevalence of overweight and obesity among Iranian children and adolescents: A systematic review and meta-analysis. Nutrition.

[37] Wang Y. (2004). Epidemiology of childhood obesity-methodological aspects and guidelines: What is new?. Int. J. Obes. Relat. Metab. Disord..

[38] Butta Z., Ahmed T., Black R., Cousens S., Dewey K., Glugliani E., Haider B., Kirkwood B., Morris S.S., Sachdev H.P.S. (2008). What works? Interventions for maternal and child undernutrition and survival. Lancet.

[39] Louw R., Bekker E., Wentzel-Viljoen E. (2001). An External Evaluation of Certain Aspects of Primary Schools Feeding.

[40] World Health Organisation (WHO) (2004). Global Strategy on Diet, Physical Activity and Health.

[41] De Ridder J.H., Coetzee D. (2013). Childhood Obesity in South Africa: Are we sitting on a time bomb?. Glob. J. Health Phys. Educ. Pedagog..

[42] National Development Plan 2030: Our Future—Make It Work. http://www.npconlineco.za/MediaLib/Downloads/Downloads/Executive%20Summary-NDP%202030%20-%20Our%20future%20-%20make%20it%20work.pdf.

[43] Strong W.B., Malina R.M., Blimkie C.J., Daniels S.R., Dishman R.K., Gutin B., Hergenroeder A.C., Must A., Nixon P.A., Pivarnik J.M. (2005). Evidence based physical activity for school-age youth. J. Paediatr..

